# Round the Hospitals

**Published:** 1916-12-02

**Authors:** 


### ROUND THE HOSPITALS.

The Hon. Arthur Stanley is indefatigable in his work for the College of Nursing. Last week he was at Liverpool, where he had one of the largest meetings of nurses ever held in that city. He invited questions and answered them with his usual completeness and lucidity. This week his engagements, we understand, include meetings at Newcastle, Birmingham, and elsewhere. This activity on his part sets a noble example for all who are genuinely interested in the success of the College. We hope, therefore, the call to action which we publish this week may be fruitful in results and workers.

It would be helpful if the active minds throughout the nursing world in the Old Country could and would ponder several types of American womanhood. Much of their secret lies in the early, thorough, and continuous training and specialised teaching to which most girls are subjected. There is in New York a school, accommodating over 5,000 girls, where the pupils do practically everything. They preside at the large assemblies of the school, they conduct debates, they administer departments and explain their work to large audiences. They display such an aptitude for the gymnasium and all muscle-developing exercises that a visitor is struck with the feeling that many of the girls at this school are equal in efficiency to the highest trained soldier of the day. In England we have something of the same system, but it is not carried out in the thorough and complete manner which is typical of American school management. At some schools they have small Zoos, so that the children may learn the habits and character of animals and get to know all the principal birds they are likely to meet with in their own country at any rate. We saw such a Zoo at a girls' school in New York, and were impressed with its condition and arrangements. All the children, and also the teachers, obtain their meals in a central restaurant, where the food is of good quality and the meals and menus are so adjusted as to suit the purses of all. Everything is served in a tasteful and appetising way, and the supplies are of the best. Cereals enter very largely into the dietary of an American household; far too largely, we should say, from the point of view of comfort, nutrition, and the after-satisfaction of eating a meal. The epidermis of an American seems to be far less susceptible, than that of the British citizen, to the skin irritability which may arise from cereal eating. The keenness of the children everywhere was remarkable, for it was a constant factor even in classes where the subjects were, to say the least, not strikingly interesting or attractive. The development of the children through the athletic exercises and drills was remarkable; but it in no case equalled the development of their teachers, which, we should say, constitutes a record in possible results from physical exercises, in women.

A meeting of the Executive Committee and members of the Association for the Promotion of Registration of Nurses in Scotland was held in the Christian Institute, Bothwell Street, Glasgow, on Friday, November 24, 1916. Lord Inverclyde occupied the chair, and, amongst others, the following members were present: Professor Glaister, Colonel Mackintosh, Major McCubbin Johnston, Dr. Newman, Sir James Affleck, Dr. Robertson (Morningside Royal Asylum), Dr. Munro Kerr, Dr. Maxtone Thom, Miss Gill, R.R.C., Miss Melrose, R.R.C., Miss Gregory Smith, R.R.C., Miss Davidson (Bangour), Miss Rough, Miss Merchant, Miss Shepherd, Miss Kay, Miss Walker, Miss Dennis, Miss Brunsey, Miss Gordon, Miss Maxwell Cameron, Dr. C. Ker, Miss Graham. Professor Glaister moved, and Miss Gill seconded, the following resolution, which was unanimously adopted and forwarded to the Central Committee meeting held on the 25th ult.: "That this Association for the Promotion of Registration of Nurses in Scotland protests against the action of the Central Committee for the State Registration of Nurses in London, in determining (1) to break off negotiations with the College of Nursing, Ltd., at a time when unanimity of action seemed so hopeful, and (2) to proceed with its own Bill without having first consulted the members of the Associations represented on the Central Committee in view of the fact that substantial progress in the negotiations between the two bodies towards an agreed Bill seems to have been achieved."

Recently the medical superintendent of a children's hospital in the country received a letter from the mother of one of the patients, asking for a photograph to be taken of her little girl, in order that she might send one to the father, a soldier, in France, who, she said, was "always thinking" about the child. In the course of a day or two the photographs were forwarded. Frequently, at the same hospital, there come from the mothers of patients requests for reports of progress, which are required in order that they may be sent to the soldier-fathers. Possibly most hospitals have had similar requests. We mention this not to suggest that the sending of photographs or special reports to patients' soldier relatives should become general, but only to show the kind of interest which links up even a children's hospital with the war. As a matter of interest to medical officers, matrons, superintendents, or sisters of hospitals where similar requests are received, it may be well to mention the existence of an organisation of amateur photographers for this very purpose of sending snapshots to soldiers serving abroad. Whether this admirable work is still being carried on we cannot say; it certainly was in full swing last year, and doubtless is so still. It seems that the members of this association might well find scope for their energies in the wards of children's hospitals, a suggestion we put forward for the consideration of all whom it may concern.

For Matrons' and Sisters' Appointments, see p. 192. For Asylum Workers see p. 191.

